# Supplementary material for: Super-elasticity at 4 K of covalently crosslinked polyimide aerogels with negative Poisson’s ratio
Source: Nat Commun. 2021 Jul 2;12:4092. doi: 10.1038/s41467-021-24388-y (PMC8253740; doi:10.1038/s41467-021-24388-y)
Supplement: Supplementary file 1 — Supplementary Information [file 41467_2021_24388_MOESM1_ESM.pdf]

Supplement Materials for

Super-elasticity at 4K of Covalently Crosslinked Polyimide Aerogels with Negative Poisson's Ratio

Yang Cheng[1, 2], Xiang Zhang[3], Yixiu Qin[4], Pei Dong[5], Wei Yao[1,2], John Matz[5], Pulickel M. Ajayan[3], Jianfeng Shen[1], Mingxin Ye[1*]*

[1]Institute of Special materials and Technology, Fudan University, Shanghai, P. R. China

[2]Department of Materials Science, Fudan University, Shanghai, P. R. China

[3]Department of Materials Science and Nanoengineering, Rice University, 6100 Main Street, Houston, TX 77005, USA

[4]State Key Laboratory of Molecular Engineering of Polymers, Fudan University, Shanghai, P. R. China

[5]Department of Mechanical Engineering, George Mason University, VA 22030, USA

[*]Corresponding Author. Email: mxye@fudan.edu.cn, jfshen@fudan.edu.cn

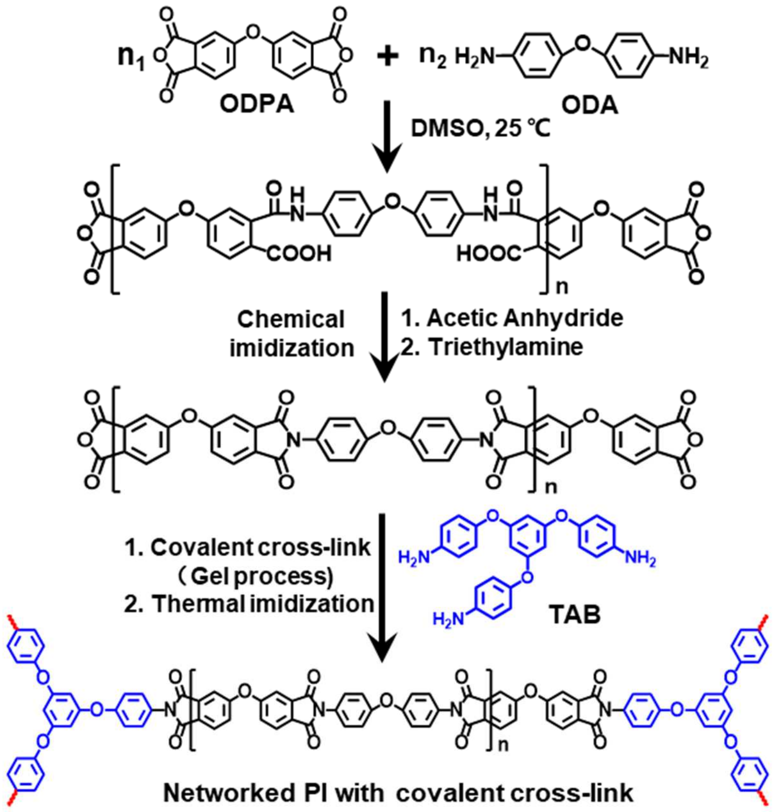

Supplementary Figure 1 Synthesis of covalently crosslinked polyimide from ODPA, ODA and TAB.

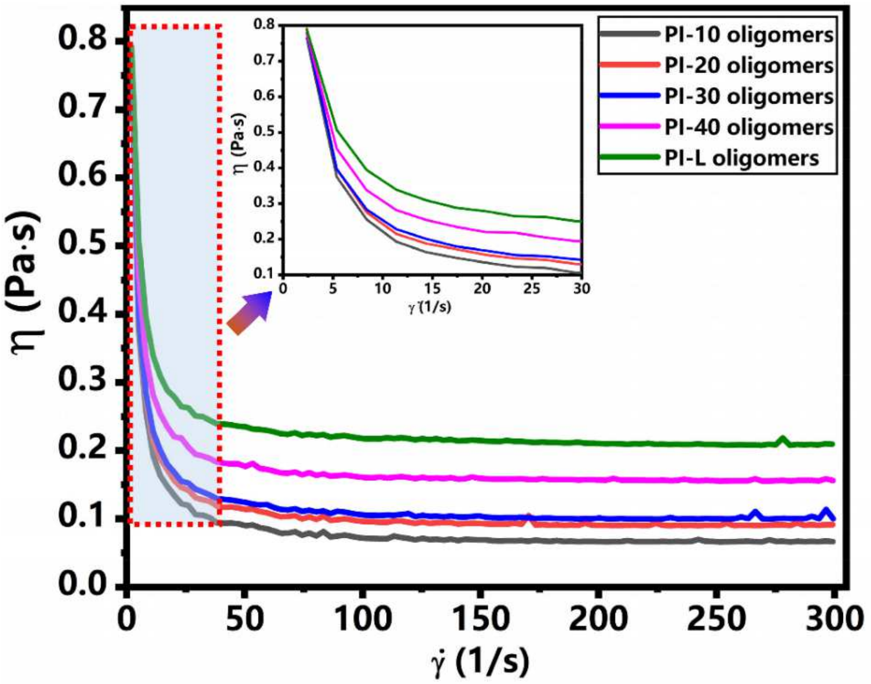

Supplementary Figure 2. Shearing viscosity curves of 6 wt% PI oligomers/DMSO solutions with different molecular weight.

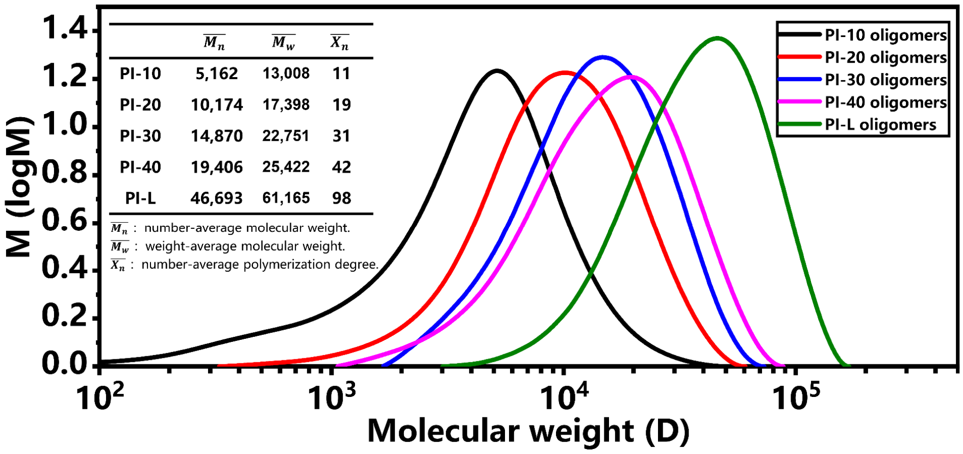

Supplement Figure 3. Molecular weight and number-average polymerization degree of PI oligomers with various polymerization degrees.

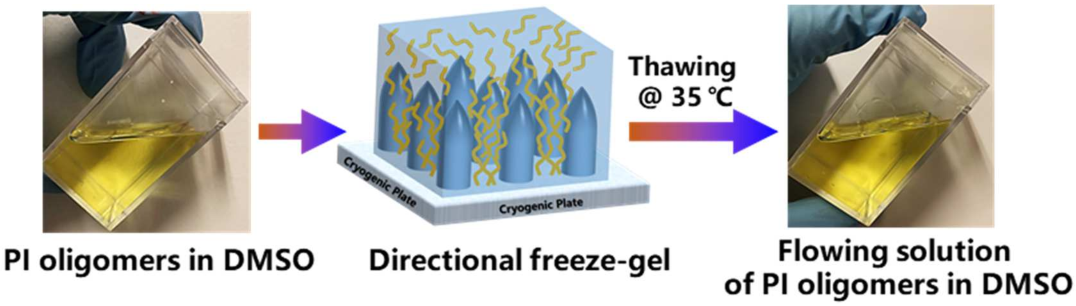

Supplementary Figure 4. Optical images after thawing frozen gel of PI oligomers without TAB.

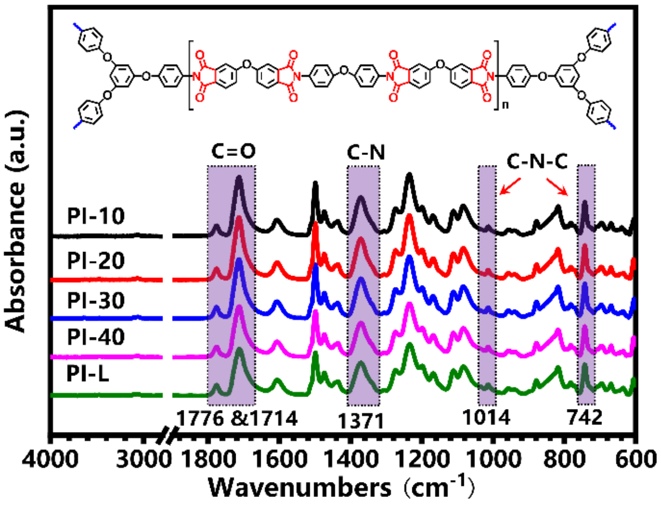

Supplementary Figure 5. FT-IR spectra of PI aerogels with different crosslinking degree.

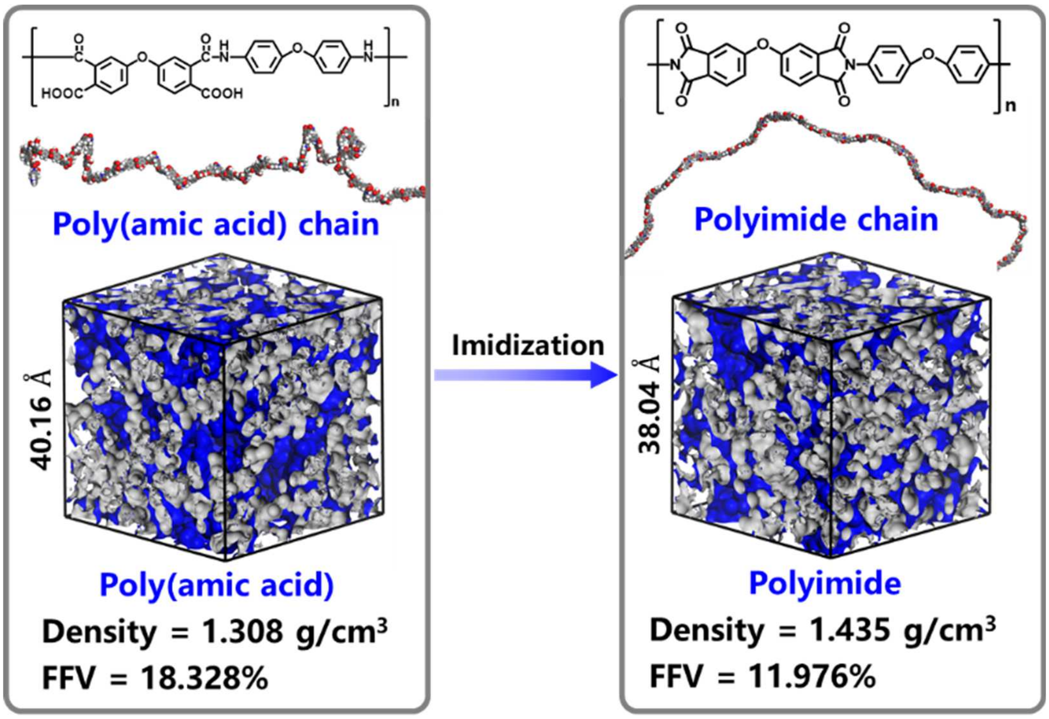

Supplementary Figure 6. Density and free volume of PAA precursor and PI simulated by Material Studio software.

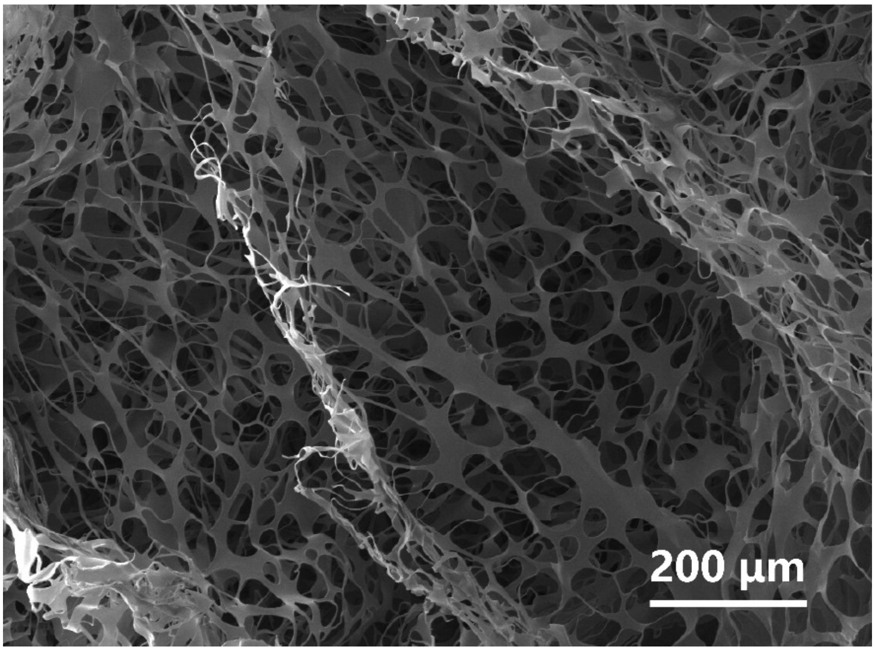

Supplementary Figure 7. SEM image of PI aerogel fabricated by ice templates assisted freeze-drying process from 1 wt% PAS aqueous solution.

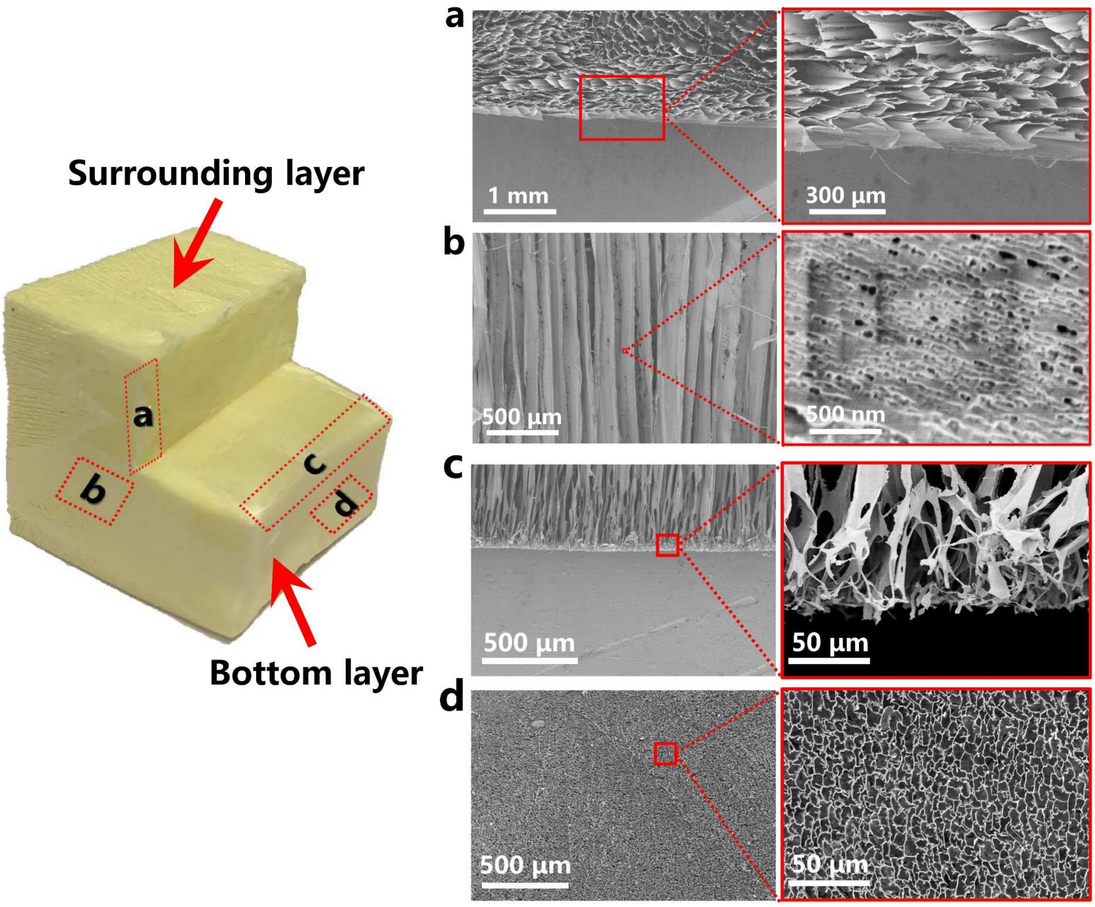

Supplementary Figure 8. Morphology of skin layers. (a) Cross-sectional SEM images of the skin layer around the aerogel. **(b)** Surface SEM images of the skin layer around the aerogel. **(c)** Cross-sectional SEM images of the skin layer at the bottom of the aerogel. **(d)** Surface SEM images of the skin layer at the bottom of the aerogel.

Supplementary Figure 8a and **Supplementary Figure 8b** show the morphology of cross section and surface of the skin layer around the PI-10 aerogel. Obviously, the skin layer is well-organized channel structure which is similar to the major architecture, revealing that the skin layer should be a horizontally periodic spread of the unidirectional architectures instead of a dense layer. In addition, mesopores (2~50 nm) could also be observed on the walls of cellular architecture as shown in **Supplementary Figure 8b**. **Supplementary Figure 8c** and **Supplementary Figure 8d** display the cross section and surface of the skin layer at the bottom of PI-10 aerogel. Although the skin layer at the bottom is denser than the major architecture, the skin layer is still a porous structure instead of a dense layer. However, the sizes of the pores (10~20 μm) at the

bottom layer are much smaller than those in major architecture (200~300 μm), which results in a visually dense layer with naked eyes. The unidirectional freeze-gelation was carried out in a mold containing uniform PI/TAB/DMSO mixture put on a freezing plate of -60 ℃. The freezing temperature at the bottom of the mold is close to the temperature of the freezing plate (-60 ℃), which is much lower than the freeze point of DMSO resulting in the formation of smaller sized DMSO crystals as a very fast freezing rate. Thus, smaller sized pores formed at the bottom of PI aerogel after freeze-drying.

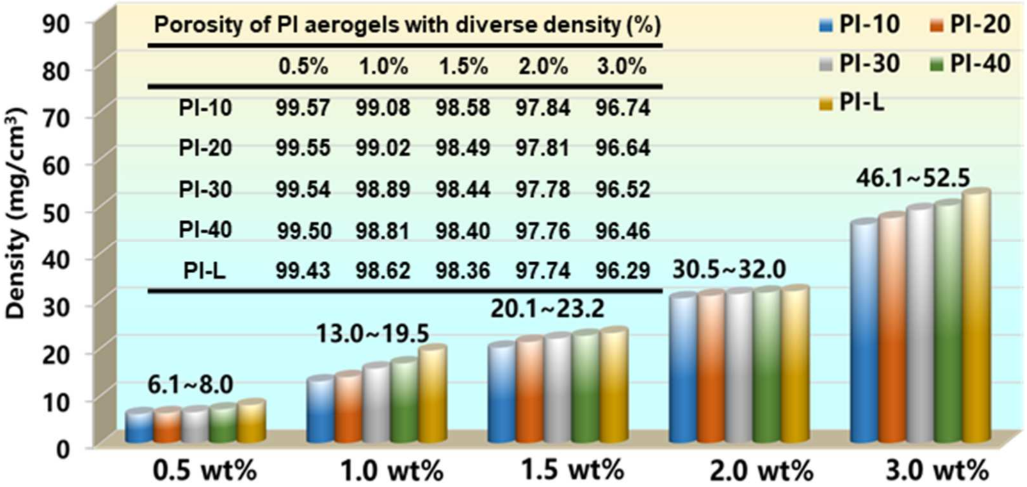

Supplementary Figure 9. Density and porosity of PI aerogels prepared from PI/TAB/DMSO with different solid contents.

$$\boldsymbol{Porosity} = \left(\mathbf{1} - \frac{\boldsymbol{\rho_A}}{\boldsymbol{\rho_{PI}}}\right) \times \mathbf{100\%}$$

Where ρ_A represents the density of aerogels, and ρ_{PI} represents the density of constituent PI ($\rho_{PI} = 1.4\ g/cm^3$).

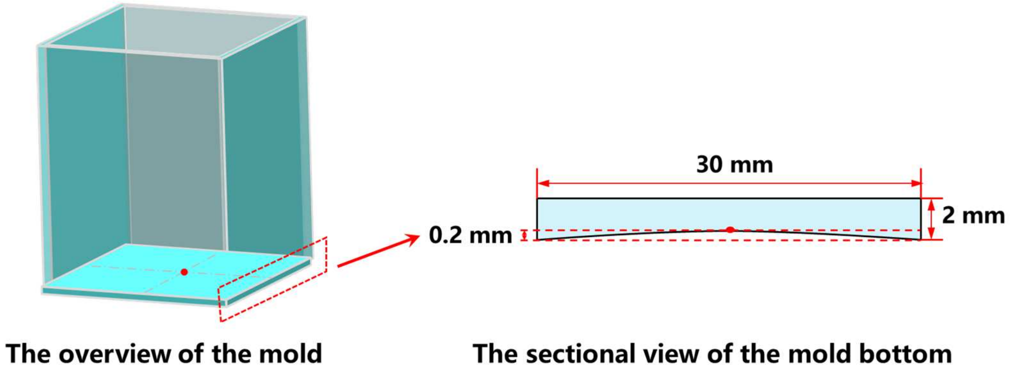

Supplementary Figure 10. Schematic diagram of the mold.

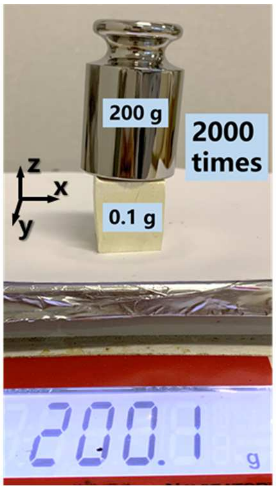

Supplementary Figure 11. Stiff properties of PI aerogel on z direction.

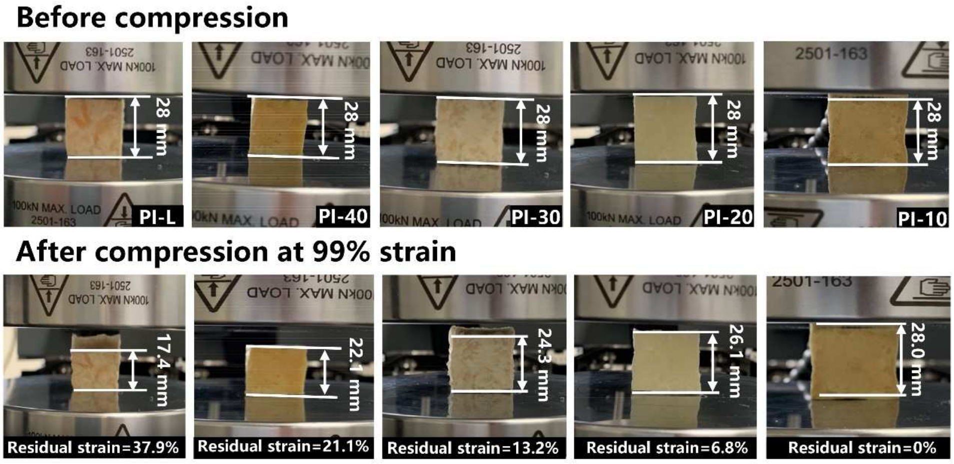

Supplementary Figure 12. Optical images of PI aerogels before and after compression at 99% strain.

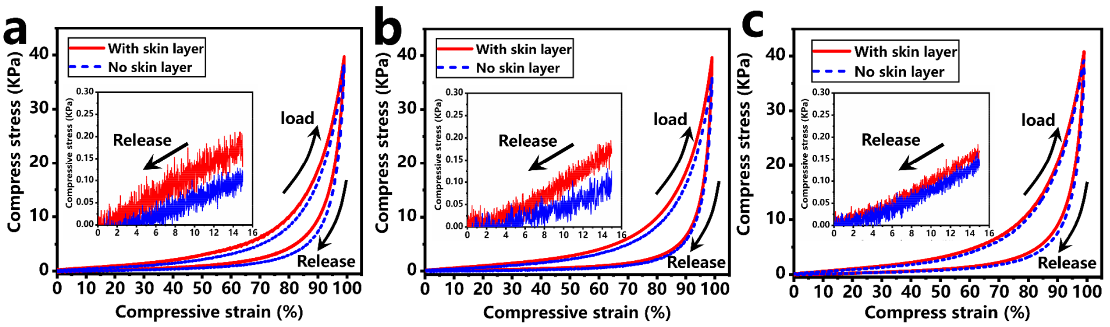

Supplementary Figure 13. Comparison investigation on stress and strain before and after removing skin layers. (a) Stress-strain curves for PI-10 aerogel with and without all skin layers. (b) Stress-strain curves for PI-10 aerogel with and without bottom skin layer. (c) Stress-strain curves for PI-10 aerogel with and without surrounding skin layer.

As shown in **Supplementary Figure 13a**, after removing all the skin layers (both surrounding and bottom skin layers), the compressive stress decreased about 3.5% (from 39.7 kPa to 38.3 kPa at 99% strain), which is close to that after removing the bottom skin layer (**Supplementary Figure 13b**). However, when the surrounding skin layer was removed, no obvious deterioration of mechanical performance was detected (**Supplementary Figure 13c**). It illustrates that the bottom skin layer improves mechanical performance owing to its smaller sized pores (more pore walls of PI) compared with the major architectures, but the surrounding skin layer has little effect on mechanical performance as it is an expansion of major architecture instead of a dense layer. In addition, through the analysis of magnifying in stress-strain curves with strain between 0~15%, we find that almost all the PI-10 aerogels can spring back to their original shape during release, whether they were removed skin layers or not. It demonstrated little impact of skin layers on ultimate resilient strain, which mainly depends on the major cellular architectures with radiational distribution and mechanical properties of covalently cross-linked PI.

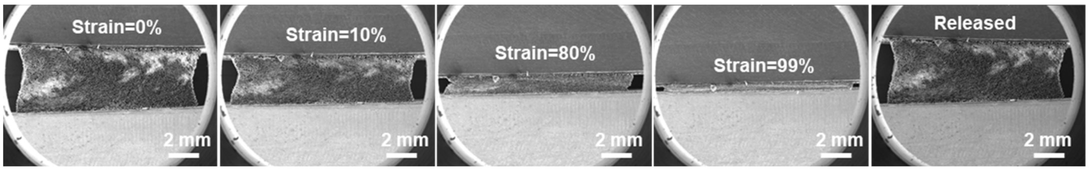

Supplementary Figure 14. Overview of PI-10 aerogels by In-situ SEM observations.

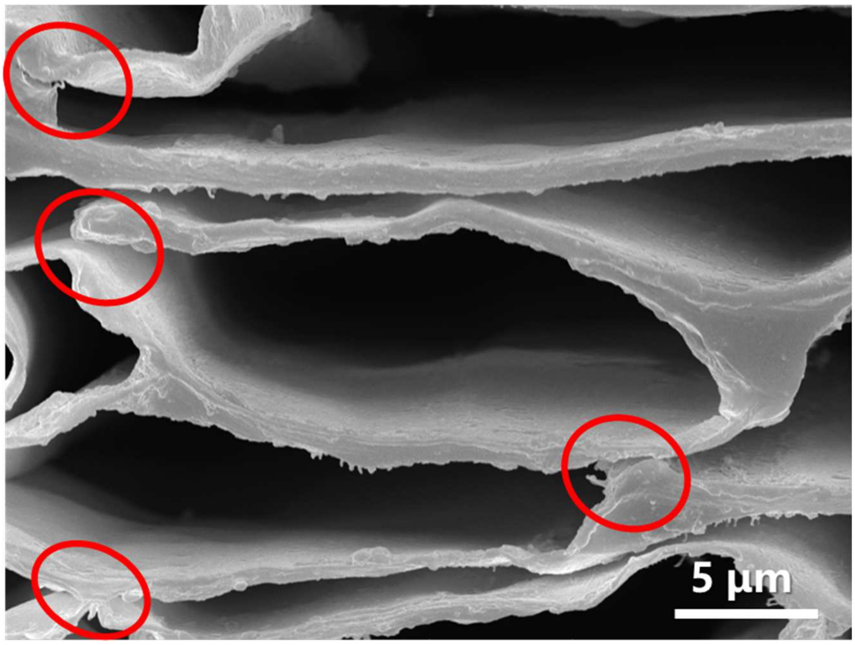

Supplementary Figure 15. SEM image of microstructure of PI-L aerogels after compression under 99% strain.

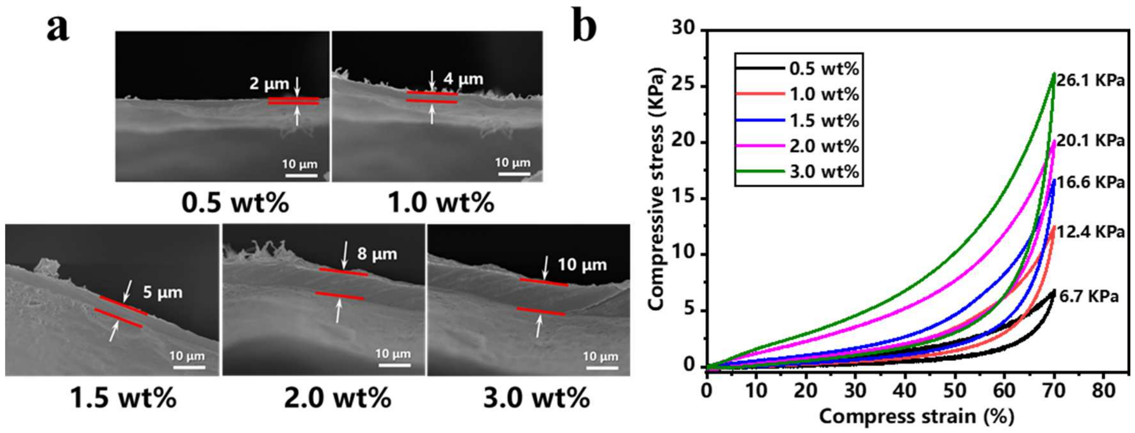

Supplementary Figure 16. Structure and resilience of PI aerogels freeze-dried from PI/TAB/DMSO solution with various solid content. (a) SEM images of PI-10 aerogels with different wall thickness, (b) Compressive stress-strain curves of PI-10 aerogels prepared from PI/TAB/DMSO mixtures with different solid content.

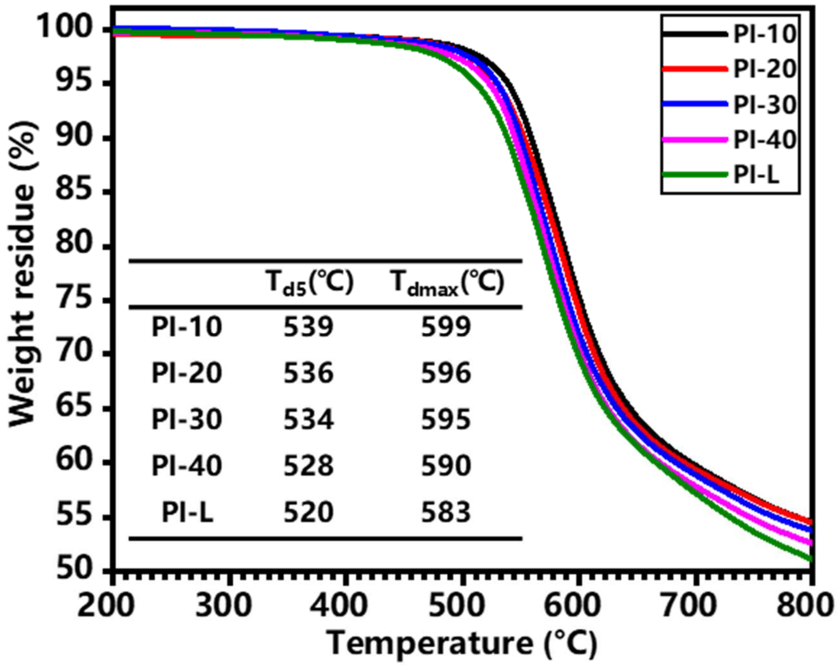

Supplementary Figure 17. TGA curves of PI aerogels with different crosslinking degree.

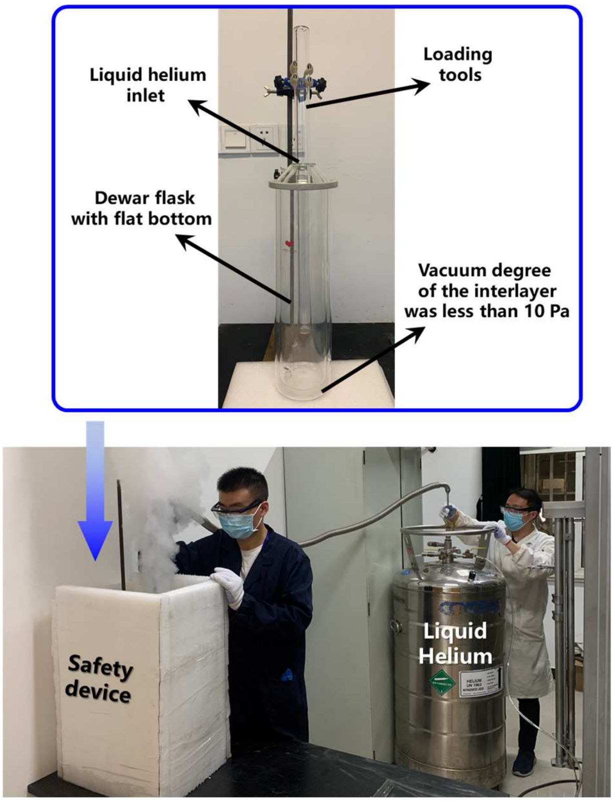

Supplementary Figure 18. Homemade apparatus for super-elastic tests of PI aerogels at 4 K.

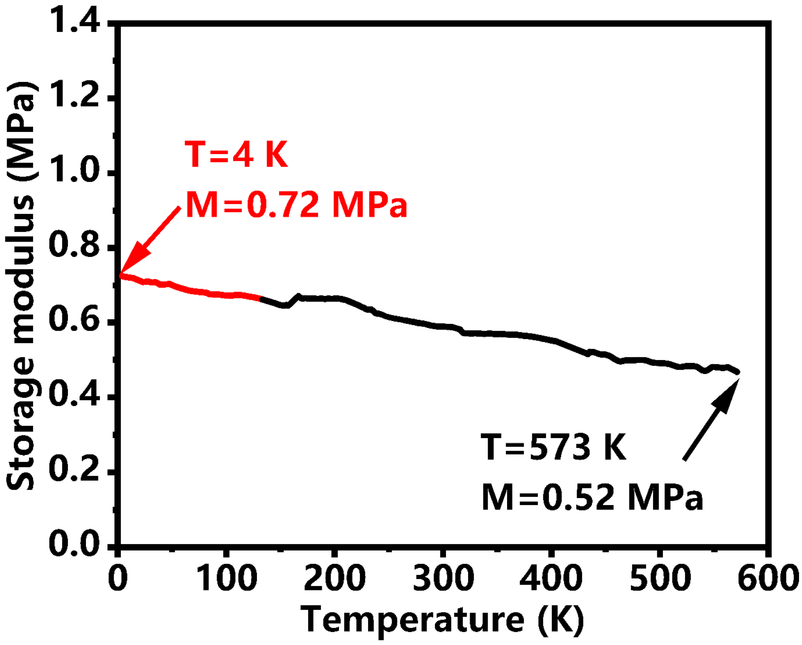

Supplementary Figure 19. Storage modulus of PI aerogel between 4 K and 573 K.

Simulated mechanical properties of PI-10 (constituent materials of PI-10 aerogels)

(1) Build model.

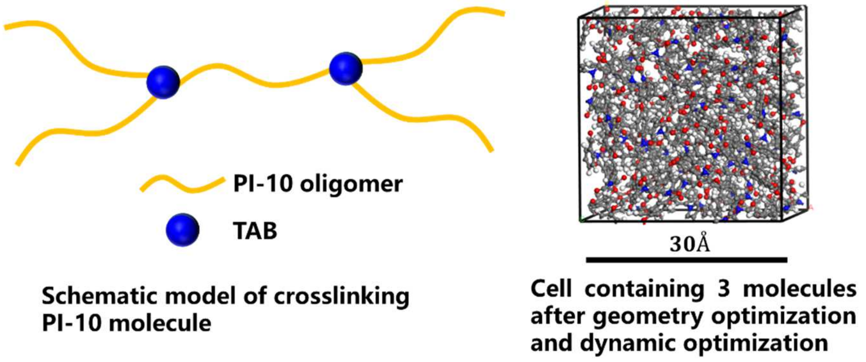

Supplementary Figure 20. Model for simulated mechanical properties of PI-10.

(2) Constant matrix of elastic stiffness and elastic compliance

PI-10 at 273 K

Elastic stiffness constants

C_{ij} (GPa)	1	2	3	4	5	6
1	6.7603	3.8101	4.1418	0.4254	0.0575	-0.3481
2	3.8101	8.2097	4.697	0.4499	-0.0889	-0.5376
3	4.1418	4.697	7.8884	0.4391	0.0005	-0.0184
4	0.4254	0.4499	0.4391	2.0261	0.0534	-0.245
5	0.0575	-0.0889	0.0005	0.0534	2.2525	0.0823
6	-0.3481	-0.5376	-0.0184	-0.245	0.0823	1.9244

Stress-i = Cij×Strain-j

Elastic compliance constants

S_{ij} (1/TPa)	1	2	3	4	5	6
1	237.0693	-56.0796	-90.2148	-14.5301	-8.8053	24.8759
2	-56.0796	203.4129	-91.0975	-8.4932	8.0617	44.3826
3	-90.2148	-91.0975	229.1450	-15.5326	0.5389	-41.5721
4	-14.5301	-8.4932	-15.5326	509.5269	-14.2429	60.3370
5	-8.8053	8.0617	0.5389	-14.2429	445.5593	-20.1955
6	24.8759	44.3826	-41.5721	60.3370	-20.1955	544.6827

Strain-i = Sij*Stress-j

PI-10 at 4 K

Elastic stiffness constants

C_{ij} (GPa)	1	2	3	4	5	6
1	10.3142	6.5583	6.1808	0.4964	-0.2515	-0.2565
2	6.5583	10.4051	6.2685	0.5836	-0.2457	-0.505
3	6.1808	6.2685	7.1694	0.402	-0.1419	-0.1987
4	0.4964	0.5836	0.402	1.602	0.1897	-0.3089
5	-0.2515	-0.2457	-0.1419	0.1897	1.6456	-0.0655
6	-0.2565	-0.505	-0.1987	-0.3089	-0.0655	1.3600

Stress-i = Cij×Strain-j

Elastic compliance constants

S_{ij} (1/TPa)	1	2	3	4	5	6
1	212.7003	-48.8503	-139.55	-15.0738	14.8721	-1.1216
2	-48.8503	219.0736	-146.632	-20.8474	16.8655	46.7947
3	-139.55	-146.632	387.3171	-4.2566	-10.3488	-25.6539
4	-15.0738	-20.8474	-4.2566	673.4025	-77.9076	138.0075
5	14.8721	16.8655	-10.3488	-77.9076	621.3477	19.7825
6	-1.1216	46.7947	-25.6539	138.0075	19.7825	780.9959

Strain-i = Sij*Stress-j

(3) Approximate calculation for shear modulus and bulk modulus

➢ Shear Modulus G (GPa)

Reuss: $G_R = \frac{15}{[4(C_{11}+C_{22}+C_{33})-4(C_{12}+C_{23}+C_{31})+3(C_{44}+C_{55}+C_{66})]}$

Voigt: $15G_V = (C_{11}+C_{22}+C_{33}) - (C_{12}+C_{23}+C_{31}) + 3(C_{44}+C_{55}+C_{66})$

Hill: $G_H = \frac{(G_V+G_R)}{2}$

➢ Bulk Modulus K (GPa)

$$\text{Reuss:}K_R = \frac{1}{[(S_{11}+S_{22}+S_{33})+2(S_{12}+S_{23}+S_{31})]}$$

$$\text{Voigt:}9K_V = (S_{11}+S_{22}+S_{33})+2(S_{12}+S_{23}+S_{31})$$

$$\text{Hill:}K_H = \frac{(K_V+K_R)}{2}$$

273 K	Reuss	Voigt	Hill
Bulk Modulus （GPa）	4.0118	4.3851	4.1985
Shear Modulus （GPa）	1.0436	1.2581	1.1509

4 K	Reuss	Voigt	Hill
Bulk Modulus （GPa）	5.1323	5.3507	5.2415
Shear Modulus （GPa）	1.8456	1.9213	1.8834

(4) Young modulus and Poisson's ratio

Young modulus: $E = 2G(1+\mu) = 3K(1-2\mu)$

273 K					
Young Modulus (GPa)		Poisson's ratio			
E_x	4.7015	μ_{xy}	0.2297	μ_{xz}	0.6561
E_y	4.5647	μ_{yx}	0.223	μ_{yz}	0.6693
E_z	2.5819	μ_{zx}	0.3603	μ_{zy}	0.3786

4 K					
Young Modulus (GPa)		Poisson's ratio			
E_x	4.2182	μ_{xy}	0.2366	μ_{xz}	0.3805
E_y	4.9161	μ_{yx}	0.2757	μ_{yz}	0.4478
E_z	4.3640	μ_{zx}	0.3937	μ_{zy}	0.3976

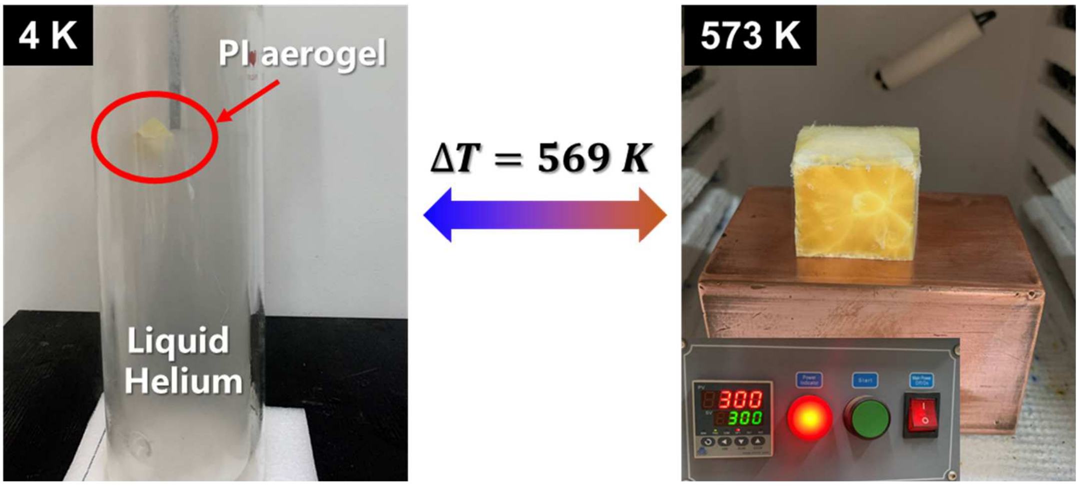

Supplementary Figure 21. Apparatus for thermal shock tests of PI aerogels.
