# Supplementary material for: Super-elasticity at 4 K of covalently crosslinked polyimide aerogels with negative Poisson’s ratio
Source: Nat Commun. 2021 Jul 2;12:4092. doi: 10.1038/s41467-021-24388-y (PMC8253740; doi:10.1038/s41467-021-24388-y)
Supplement: Supplementary file 3 — Description of Additional Supplementary Files [file 41467_2021_24388_MOESM3_ESM.docx]

**Description of Additional Supplementary Files**

Title: Supplementary Movie 1.

Description: Formation of DMSO crystals at initial stage of freeze gelation.

Title: Supplementary Movie 2.

Description: Bending performance of PI aerogel.

Title: Supplementary Movie 3.

Description: Compression and recovery between 0% and 99%.

Title: Supplementary Movie 4.

Description: Super-elastic performance in liquid helium (4 K).
